# “You do it for the patient”: a qualitative analysis of changes to primary care nurses’ workplace demands and resources during the COVID-19 pandemic in Canada

**DOI:** 10.3389/frhs.2025.1557654

**Published:** 2025-06-26

**Authors:** Sarah Spencer, Lindsay Hedden, Julia Lukewich, Emily Gard Marshall, Maria Mathews, Samina Idrees, Jennifer E. Isenor, Ruth Martin-Misener, Leslie Meredith, Crystal Vaughan, Dana Ryan

**Affiliations:** ^1^ Faculty of Health Sciences, Simon Fraser University, Burnaby, BC, Canada; ^2^ Faculty of Nursing, Memorial University, St. John’s, NL, Canada; ^3^ Department of Family Medicine, Faculty of Medicine, Dalhousie University, Halifax, NS, Canada; ^4^ Department of Family Medicine, Schulich School of Medicine & Dentistry, Western University, London, ON, Canada; ^5^ College of Pharmacy, Faculty of Health, Dalhousie University, Halifax, NS, Canada; ^6^ School of Nursing, Faculty of Health, Dalhousie University, Halifax, NS, Canada

**Keywords:** primary care, nurses, Canada, pandemic, burnout, wellbeing

## Abstract

**Introduction:**

Primary care in Canada was an essential component of the COVID-19 pandemic response, as well as continued provision of routine care. Yet, primary care settings were inadequately supported during the pandemic, leaving clinicians feeling vulnerable and overwhelmed. Existing pandemic research has focused on the health workforce broadly or those working in acute care settings. Accordingly, we sought to understand the personal and professional experiences of nurses working in primary care settings in Canada during the COVID-19 pandemic to inform future pandemic responses and health workforce planning that account for primary care nurses’ mental and physical needs.

**Methods:**

We conducted semi-structured interviews with licensed and registered practical nurses, registered nurses, and nurse practitioners working in primary care in four Canadian provinces: British Columbia, Newfoundland and Labrador, Nova Scotia, and Ontario. Interviews were recorded, transcribed, and thematically analyzed.

**Results:**

We interviewed 76 primary care nurses about their pandemic experiences. Using the National Academy of Medicine's systems model of clinician burnout and professional wellbeing, we categorized participants’ experiences according to their job demands and job resources. These data describe how COVID-19 altered primary care nurses’ professional experiences across a variety of areas, often with implications for their wellbeing.

**Discussion:**

Prior to the pandemic, primary care nurses could rely on their job resources to protect against the demands they regularly encounter; however, many of these resources were negatively affected by the pandemic. Improved pandemic preparedness, including primary care-specific supports to promote physical and psychological safety, workflow efficiency, worker rest and recovery, and preservation of primary care capacity are needed to uphold primary care nurse wellbeing during a pandemic or other extended health emergencies.

## 1 Introduction

Canadian policy initiatives have increasingly focused on the expansion of interprofessional primary care teams that include integral roles for nurses (1–4). There are three main designations of nurses working in primary care in Canada: licensed practical nurses (LPNs) [referred to as registered practical nurses (RPNs) in Ontario]; registered nurses (RNs); and nurse practitioners (NPs). Primary care nurses (PCNs) fill a critical role in the delivery of primary care, with each nursing designation carrying a different legislated scope of practice that informs the care they are able to provide (5).

During the COVID-19 pandemic in Canada, primary care was essential to the pandemic response (e.g., surveillance, education, community management) as well as continued provision of routine primary care (6–10). Despite this integral role, research focusing on primary care experiences during the COVID-19 pandemic indicates that primary care workers and settings were inadequately supported—with the focus instead placed on the health workforce broadly or those working in acute care settings (7, 11–14). This lack of support—ranging from timely COVID-19 updates and practice guidance to appropriate personal protective equipment (PPE)—left primary care clinicians feeling vulnerable and overwhelmed (15–19). These concerns could be exacerbated for clinicians who feared passing on any workplace exposures to their children, elderly parents, or immunocompromised people in their lives (15, 17, 19, 20). Primary care providers reported increased work hours, heightened feelings of stress, and a lack of resources relative to other health settings, contributing to increased burnout and decreased job satisfaction (13, 21–26). PCNs, specifically, have reported distress stemming from their pandemic workload, experiences of social animus, and the risks of COVID-19 exposures (27, 28). Halcomb and colleagues found, through their survey of Australian PCNs, that almost half of the respondents were experiencing some degree of depression, anxiety, or stress which participants attributed to the COVID-19 pandemic (16).

Clinician wellbeing is a crucial element of a robust health system and particularly important during health emergencies and periods of high stress, and necessitates that healthcare professionals are not working to their mental and physical limits for prolonged periods (11, 29). Yet, existing research amongst primary care professionals internationally (11–14, 21) and in Canada (18, 26) suggests that resources to support clinician wellbeing are not commonly provided. Much of this research, however, has focused on healthcare workers broadly or those working in hospitals (30); there is little research on the experiences of PCNs in Canada to help understand their experiences and their associated wellbeing during and beyond the COVID-19 pandemic. Accordingly, our objective is to explore the personal and professional experiences of nurses working in primary care settings in Canada during the COVID-19 pandemic to inform future pandemic responses and health workforce planning that account for PCNs’ mental and physical needs.

## 2 Methods

### 2.1 Study design

As part of multiple case study, we conducted semi-structured interviews with PCNs across four Canadian provinces: the Interior, Island, and Vancouver Coastal health regions of British Columbia (BC); the province of Newfoundland and Labrador (NL); the province of Nova Scotia (NS); and the Ontario Health West region in Ontario (ON). These interviews explored the roles, responsibilities, and experiences of PCNs during the COVID-19 pandemic. This specific analysis focuses on PCNs’ personal and professional wellbeing experiences, as part of a broader research project undertaken to identify and describe the anticipated and actual roles of primary care professionals during the COVID-19 pandemic in Canada (31).

### 2.2 Sampling and recruitment

Using a purposeful maximum variation sampling approach, we recruited participants with diverse characteristics, including gender identity, community size, number of years in practice, practice models (e.g., collaborative team clinics, NP-led clinics, private practices), and clinic funding models (e.g., fee-for-service, alternate payment plans).

Eligible participants included LPNs/RPNs, RNs, and NPs who were clinically active or eligible to be clinically active and who worked in primary care during the COVID-19 pandemic (i.e., January 2020 through the interview period). We excluded PCNs who worked exclusively in administrative, academic, or research positions and nursing students who were practicing in a clinical placement or preceptorship.

Members of our research team distributed study materials using formal and informal nursing networks (e.g., nursing professional organizations, academic lists, public listings, health authorities), newsletters, social media, and snowballing [where permitted by regional ethics boards (i.e., BC, NL, NS)]. Principal investigators and research assistants shared invitations and study information with prospective participants, answered any questions, and obtained written (by email or fax in advance) or recorded verbal (at time of interview) informed consent.

### 2.3 Data collection and analysis

We conducted interviews by phone or Zoom (Zoom Video Communications Inc.), based on participant preference, between May 2022 and January 2023. Interviews explored the varied roles of PCNs as the pandemic evolved, facilitators and barriers PCNs encountered, demographic and practice characteristics, as well as any gendered and personal experiences. Our interdisciplinary team created and pre-tested the interview guide (Supplementary File S1). Interviews ranged from 23 to 125 min (average 57 min) in length and were audio-recorded, transcribed verbatim, de-identified, and verified by the interviewer.

We analyzed interview transcripts using a descriptive thematic approach (32) using a harmonized codebook developed collaboratively by experienced qualitative researchers on the study team. To develop this codebook, one researcher from each regional team inductively coded an interview from their study region to create a preliminary codebook. These researchers then applied their regional codebooks to a pre-determined sample of transcripts representing each region and nursing designation (LPN/RPN, RN, NP) before meeting to discuss coding decisions and develop a harmonized codebook with consistent labels and descriptions. The process continued until all members of the coding team were satisfied with the completeness of the codebook and the consistency of code application. Regional researchers then used the final harmonized codebook to code all transcripts from their region using NVivo (QSR International).

**Figure 1:**
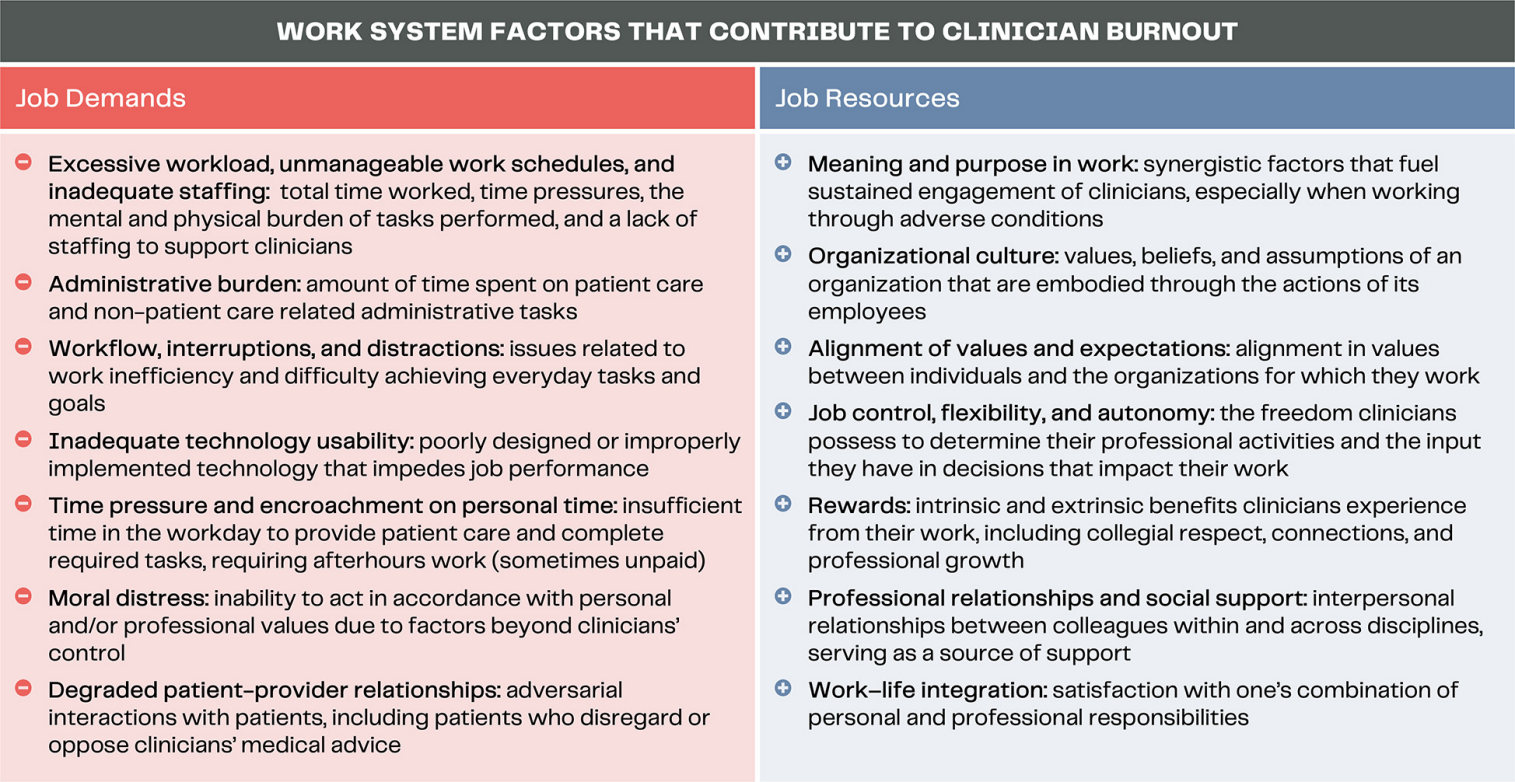
Work system factors of the National Academy of Medicine's systems model of clinician burnout and professional wellbeing [adapted from (29)].

For the analysis presented in this manuscript, we analyzed data previously coded under Nurse Mental Health and Wellbeing, Risk Navigation, Personal and Professional Tensions, and Personal and Professional Supports. We then employed the American National Academy of Medicine's systems model of clinician burnout and professional wellbeing (29) to deductively analyze and organize this subset of data. This framework (Figure 1) identifies the ‘job demands’ and ‘job resources’ as perceived by the individual that, respectively, can contribute to or protect against clinician burnout. Job demands encompass the “physical, social or organizational aspects of work that require a certain mental or physical effort and a consequent expenditure of psychological or physical energy” (33). Whereas, job resources are the “physical, psychological, social, or organizational aspects of work that can: be functional in achieving objectives; reduce job demands; stimulate personal growth and professional development” (33).

### 2.4 Ethics and study rigor

Regional study teams obtained approval from their relevant ethics boards: harmonized Research Ethics of British Columbia, the Health Research Ethics Board of Newfoundland and Labrador, the Nova Scotia Health Authority Research Ethics Board, and Western University Research Ethics Board. All participants provided their informed consent prior to their interview. We anonymized study participants and report direct quotes using unique participant codes.

We are an interdisciplinary team of primary care researchers and experts, including nurses, public health officials, and policymakers and we adopted a pragmatic approach to conducting this research (32, 34, 35). We validated participant responses by checking meaning during interviews conducted by experienced interviewers (36–38). We looked for negative cases throughout analyses and present results using thick, participant-centered descriptions (38).

## 3 Results

We interviewed 76 PCNs between May 2022 and January 2023, with 94.7% of participants self-reporting as women and 65.3% reporting dependents and/or personal caregiving responsibilities (Table 1). Participants’ pandemic experiences are grouped according to the work system factors of the systems model of clinician burnout and professional wellbeing described above (29). Aligning with the factors outlined in the National Academy of Medicine's framework, the data portrays changes to PCNs’ (1) job demands and (2) job resources during the pandemic, specifically focusing on those that may have affected their wellbeing.

**Table 1:** Participant characteristics by study region [*N* (%)].

Characteristics	BC *N* = 13 (17.1)	NL *N* = 16 (21.1)	NS *N* = 20 (26.3)	ON *N* = 27 (35.5)	Total *N* = 76 (100.0)
Regulatory designation					
LPN/RPN	0 (0.0)	5 (31.3)	1 (5.0)	9 (33.3)	15 (19.7)
RN	11 (84.6)	6 (37.5)	11 (55.0)	9 (33.3)	37 (48.7)
NP	2 (15.4)	5 (31.3)	8 (40.0)	9 (33.3)	24 (31.6)
Gender^a^					
Man	–	–	–	–	3 (3.9)
Woman	–	–	–	–	72 (94.7)
Non-binary	–	–	–	–	1 (1.3)
Dependents^b^					
Yes	9 (69.2)	10 (62.5)	14 (70.0)	17 (63.0)	50 (65.8)
No	4 (30.8)	6 (37.5)	6 (30.0)	10 (37.0)	26 (34.2)
Community size^c^					
Rural	1 (7.7)	6 (37.5)	11 (55.0)	10 (37.0)	28 (36.8)
Small urban	3 (23.1)	0 (0.0)	6 (30.0)	5 (18.5)	14 (18.4)
Urban	9 (69.2)	9 (56.3)	3 (15.0)	12 (44.4)	33 (43.4)
Mixed^d^	0 (0.0)	1 (6.3)	0 (0.0)	0 (0.0)	1 (1.3)
Practice setting					
Community	10 (76.9)	15 (93.8)	20 (100.0)	19 (70.4)	64 (84.2)
Hospital	0 (0.0)	0 (0.0)	0 (0.0)	1 (3.7)	1 (1.3)
Rural Health Center	3 (23.1)	0 (0.0)	0 (0.0)	2 (7.4)	5 (6.6)
Other^e^	0 (0.0)	1 (6.3)	0 (0.0)	5 (18.5)	6 (7.9)
Years in practice [Mean (SD)]					
Current Designation	17.7 (11.2)	14.0 (9.8)	15.6 (11.3)	13.6 (10.1)	14.9 (10.3)
Redeployed during COVID-19					
No	4 (30.8)	9 (56.3)	9 (45.0)	19 (70.4)	41 (54.0)
Yes	9 (69.2)	7 (43.7)	11 (55.0)	8 (29.6)	35 (46.0)

Abbreviations: BC, British Columbia; NL, Newfoundland and Labrador; NS, Nova Scotia; ON, Ontario; LPN/RPN, licensed/registered practical nurse; RN, registered nurse; NP, nurse practitioner.

^a^ Gender was asked as an open-ended question. We have grouped genders across all study regions to maintain confidentiality due to our small sample size of non-binary participants.

^b^ Dependents includes children, parents, and other family/community members for whom participants identified being responsible.

^c^ Rural <10,000 population, small urban = 10,000–99,999 population, urban >1,000,000.

^d^ Mixed represents participants who reported practicing across differently sized communities.

^e^ ‘Other’ practice settings include academic clinics, home and community care, public health, and private corporations.

### 3.1 Job demands

#### 3.1.1 Excessive workload, unmanageable work schedules, inadequate staffing

Participants routinely described how their workload shifted during the pandemic. For many PCNs, the pandemic brought with it a new set of demands (e.g., infection prevention and control protocols, staying up to date on rapidly evolving COVID-19 guidelines) that were added to their existing workload:

…many [PCNs] are already run off their feet. How thin can you spread them without actually starting to compromise the health that you're trying to help? If they're too busy to do one, if you're too busy to do three or four things, how are you going to add a fifth and still be able to do the job properly? Like when [are] things going to start to fall through the cracks because things have been spread so thin? [NS04-NP]

For many, the increase in workload meant longer hours and more consecutive days worked: *“I was working seven days a week, 15 hours a day”* [BC08-RN]. Many participants felt equipped to handle this schedule initially, but not in the longer-term:

It was like a year-and-a-half … I was so worn down—because my husband kept saying, ‘You got to take some time off, you're working too hard, you're working too much.’ And I was like, ‘Well, no, I feel good. As long as I feel good, I'm just going to keep going.’ But … that wasn't a smart thing. And then, finally, it just hit me and I had kind of an emotional breakdown. [ON22-NP]

When asked what resources PCNs needed, a consistent response from participants was simply *“more staff”*, recognizing that “*everybody needs help, right?”* [ON20-NP]. PCNs specifically wanted more nursing help, appreciating their utility and suitability for the work that was needed: “*So, more staff certainly would have been helpful, and I think more nursing staff would have been appropriate for a lot of the … [extra tasks] that we were just sort of worried about”* [NS02-NP].

The calls for more staff—nurses in particular—were echoed in PCNs’ feeling unable to take time off given their patients’ needs and the lack of coverage available:

It’s difficult because what nurses … need the most is [the] ability to take time off when they do need to recharge or refresh or do something for their family … You can't get time off because of the short staff. I have over 150 vacation hours from last year that I didn't get to take and I’ll have that many again. … It’s hard to be willing to take [time off] when people are so desperate for care. [NS08-NP]

#### 3.1.2 Administrative burden

Further, several participants remarked how the onslaught of emails with COVID-19 updates became a source of frustration: “*I will tell you what they needed to stop doing was stop sending fucking stupid emails on a Friday night at 5 o’clock when no one's in the hospital to support”* [ON01-RN]. Some PCNs expressed a desire for their managers to provide a synopsis of the emails, since the volume of updates became untenable in conjunction with their clinical workload:

…the Health Authority that I work for, they were able to provide email updates. My concern with those is you're already so stretched thin that sometimes it was really hard to actually read them all. … It would have been more helpful from our team leader perspective … to actually have them do a quick synopsis and say, ‘This is how COVID impacts you this week, this is the new information’… [NS07-NP]

Another PCN detailed the administrative process they encountered when they had to isolate after a COVID-19 exposure, reflecting discordance in policies that added unnecessary stress:

I had such a battle. … You're telling me I have to stay home for seven days, but yet you're telling me I need [a functional assessment form] because I'm off more than five days. … I was resentful over that. And I refused, I am not going and paying money to get a functional assessment form done when the guidelines are telling me I have to stay home for seven days … [NL19-RN]

#### 3.1.3 Workflow, interruptions, and distractions

With COVID-19 introducing immense uncertainty, many PCNs faced a complete overhaul of their work: “*we had to learn how to do things differently”* [ON21-RPN]. For some participants, the pandemic saw their entire workflow shift due to redeployments, whether mandated or voluntary. The advent of COVID−19-specific programs required instant adaptation, as detailed by an NP from ON:

Once the COVID@Home Monitoring Program [virtual visit program for following COVID-positive patients] started, that’s kind of when everything changed in terms of day-to-day workflow. I had to … reduce my role in the same-day clinic … to be able to manage everything. [ON17-NP]

For other PCNs, their workflow shifted within their regular clinic setting. As some healthcare professionals minimized their in-person interactions, PCNs who continued to see patients in-person had to modify their traditional processes:

So, [other healthcare professionals] were completely protected compared to us, where we were in the room, being exposed, and then also juggling all these roles. And even disruption of paperwork and getting orders—… they would make orders at the bedside, and you would transcribe them and initiate things right away. But there’d be a delay in care because you've got your neonatologist on a different floor in a room somewhere else on a camera who you can kind of hear and you have to wait until the orders come down. So, it really changed the workflow. [NS07-NP]

#### 3.1.4 Inadequate technology

The rapid expansion of virtual primary care had varied effects for PCNs. Some participants wanted to provide care remotely, particularly as it could reduce their exposure risks and allow them to be home with children while still working. But some PCNs encountered barriers to providing care virtually, as was the case with one RN who was told they did not have the right technology to provide virtual care:

…initially, I was told that I could not work from home because I didn't have a Health Authority assigned phone. … I eventually did work from home, but that took…over a year into the pandemic. So that was hard because I was very anxious about coming into the office, even though I wasn't really seeing people. I have two young kids at home and my husband also works in healthcare, so, just leaving the house every day was really hard. [NS11-RN]

Other PCNs noted the challenges they encountered with videoconferencing technology. For some, these challenges were tied to navigating different virtual care platforms and knowing which service providers were using which program, which introduced new obstacles for PCNs coordinating patient care:

…sometimes I'll call certain [specialist’s] offices and they'll be like, ‘oh, we don't use that anymore. We use [a different virtual platform], or we use this, and we…,’ you know, now there’s so many different things… [ON27-RNP].

Collectively, PCNs’ experiences with virtual care during the pandemic could introduce usability barriers, contributing to their already challenging workloads and anxieties around COVID-19 exposure.

#### 3.1.5 Time pressure and encroachment on personal time

The pandemic negatively impacted PCNs’ personal time. For those PCNs who had the ability to work remotely, this could impact their approach to administrative tasks. As participants noted, the lack of separation between work and home often resulted in them catching up on charting in the evenings at home instead of in their clinic the next workday.

More broadly, participants noted the long hours and increased workload that they faced amidst the additional work required by the COVID-19 updates and procedures:

… we were just expected to carry on, even with all of the additional time components. So, at the end of the day when you're not being compensated for the additional hours, but because you care and you've got this information and you want to see the patient do well, you do these things. But then you're also expected to read these emails and updates and watch these videos and things were ever-changing. [NS07-NP]

As one single parent described, their pandemic schedule affected their ability to perform some of their regular household roles:

I would be getting home sometimes 12 or 1 in the morning and then getting up and being back at it, 6, 7, 8 o’clock in the morning and gone again for the full day. So, you know, [the kids] had to pack their own lunches for school and get their own way to school and I definitely needed support from other people for driving them and so on. [NL13-RN]

These schedule changes could be more than just exhausting for PCNs; the absence from their families was also emotionally challenging: “*I felt guilty that I was at work and not home … it was difficult for me to see all of my friends spending the quality time with their kids either working from home or cutting down on their hours”* [NS01-RN]. Alternatively, some PCNs with young children were required to shift their clinic hours so that they could support homeschooling and provide childcare: “*I did have to move my clinic shifts, so, I did evening shifts and more weekends, which didn't lend itself to having much time off*” [NL14-NP].

Other PCNs noted how the public health measures enacted and their role as a healthcare professional meant that their life became all about work, whether consciously or not:

Well, I had no life—I would say I live at work and I visit my house sometimes. … It was just work and home, home and work. And it took a toll because that’s all you do. You know, COVID 24/7 and I got so bad that I was even dreaming about cases. And there wasn't a break. So, even when I'm sleeping, I couldn’t get a break because I’m still thinking about cases … and if we dare take a day off, then what happens? All of our 20 cases … and then you feel bad because you're putting more work on the nurses that are left behind and you don't want to do that because you know what it’s like. [ON06-RN]

While dreams about patient cases were not recurrent across our interviews, the pandemic found other ways to intrude on PCNs’ personal time. Participants frequently recounted how they had become an unwilling resource for family and friends, providing education and support on how to navigate the pandemic: “*I do not profess to being a COVID expert, I am not an epidemiologist—but all my friends, family members and everybody was constantly like, ‘what should we do?’* [ON25-RN].

#### 3.1.6 Moral distress

PCNs routinely detailed the distress they experienced themselves and that they witnessed amongst their colleagues. Some of these experiences were related to the risks they faced personally, or the potential risks they were introducing to their household by their work:

I will say that it is the one and only time that I've ever remembered in my [35+ year] career where I saw so many of my colleagues have so much fear. … where they had to make decisions to care at a time that could hurt them or kill them or hurt their families. [NS01-RN]

In other cases, PCNs noted the guilt they experienced due to the precautions they took to protect family members, particularly with young children who could not comprehend the rationale: “*So, you know, when you have small children, how are you going to explain to them you are not holding them as usual because you want to prevent a contact or an infection?”* [ON24-NP].

Many participants noted how they felt compelled to work throughout the pandemic, the feelings of guilt if they had to isolate due to an exposure or illness, and how this could be compounded by the experience of notifying their employer or patients:

Don’t make people feel guilty when they’re sick. I have huge moral distress if I have to call in sick, huge moral distress because I know that my next available clinic day is four weeks out and these people have already been waiting four weeks. So, it’s eight weeks for primary care … So, I never want to call in sick. So, I had COVID last week and what did I do? Work from home. [BC13-NP]

Other participants detailed how patient care experiences became a source of distress during the pandemic. Pandemic precautions, such as those that limited the number of individuals who could attend a clinic visit or the ability for family members to see inpatients, were especially challenging for PCNs. The healthcare experiences of individuals, such as those in long-term care facilities [e.g., “*As a nurse, I'm very discouraged in what happened to our elderly”* (ON01-RN)], were also a source of distress for some participants; so too were patient outcomes [e.g., “*we had a 30-year-old patient who did not get her COVID vaccine and died from COVID. And left her 9-year-old son”* (ON11-RPN)].

#### 3.1.7 Degraded patient-provider relationships

Participants noted how the pandemic altered their existing relationships with patients. Frequently, PCNs recalled frustration with patients’ non-disclosure of symptoms during COVID-19 screenings so that they could see a clinician, placing nurses at risk for infection*: “not every patient reports their symptoms, right? They want to be seen for something else, so, they'll say, ‘No, I don't have COVID’.”* [NS02-NP]. Participants also discussed how, most notably once vaccines became prominent, some patient interactions became particularly draining:

If somebody starts talking about conspiracy theories and they start yammering on about B.S., I have to sit there and smile and not say nothing because of course I'm not going to get into a fight with them … as a nurse, you can't say, ‘Well, I…’ and ‘Me, I think…’ you have to always maintain professional. You try to enlighten them … the vaccines are safe, but no matter what … I can't change their mind. [ON16-RN]

Some patient interactions also became confrontational, particularly for PCNs tasked with notifying patients that they needed to isolate after an exposure or positive COVID-19 test: “*having to call somebody and tell them they have to quarantine for 14 days … and that person saying they wouldn't be able to have an income, or they would be quarantined from their loved ones. … [you have to be] prepared for very angry outbursts”* [ON08-RN].

### 3.2 Job resources

#### 3.2.1 Meaning and purpose in work

Amidst the upheaval, exhaustion, and challenges PCNs faced during the pandemic, many participants expressed that this was the nature of their profession: “*I think that’s why most nurses are nurses. They are in it to help people … they're not martyrs, but they're people who just want to do good and help people … you do it for the patient”* [NS06-NP]. For some PCNs, the purpose behind their work helped them to keep going: “*I felt valued and I knew what I was doing was helping. So that felt good and I think that kind of powered me through”* [ON22-NP]. One participant even welcomed the heavier workload, as it and the care they were providing served to distract them from some of the fears and uncertainty that COVID-19 introduced:

I personally do struggle with anxiety … I’m not sure that the pandemic made it worse, it might have made it a bit better … we were so focused on trying to do the best for people that I didn’t really have time to focus on being anxious. [ON25-RN]

Some PCNs, depending on their clinic setting and roles during the pandemic, even felt they were not doing enough to contribute to the pandemic response. As one participant detailed, volunteering in the community helped address their feelings of not contributing to the pandemic response in their professional life:

I had so much guilt around that, that my brothers and sisters in nursing, so many were on the frontlines working during the pandemic and I thought, I’m not there and I’m not helping and I’m not doing enough. And then … it was in the news that they were anticipating that our seniors were really going to suffer during the pandemic because so many lived alone and they were going to be isolated … so, I called and I signed up to be a volunteer and it started with just weekly phone calls to an elderly lady who lived all by herself. We talked on the phone for over a year and … it was a way for me to feel like I was contributing to help during the pandemic. [BC04-RN]

Other participants echoed this sentiment. Despite conflicting feelings about leaving their kids to provide frontline clinical care during the pandemic, one NP in ON felt that it was their responsibility as a nurse to help their community:

…just being a nurse … I need to be helping, I need to be doing. And it was almost like the competing emotions, where … I want to be home and be safe with my kids, and then … I need to help and get out and use my skills. [ON22-NP]

#### 3.2.2 Organizational culture

The settings in which and the people with whom PCNs worked strongly influenced their pandemic experiences. While this was not the case for all participants, some indicated that their clinic team helped make their pandemic experience less arduous:

…there’s not anyone here that wouldn't walk by a needle bucket and change it, even though it’s not ‘their job’. No one has a job that’s not ‘their job’ here, and I think that does make a difference. … and that’s why we're able to make changes quick … We just go, ‘look there’s a problem here; what can we do?’ And everybody jumps in to try and help fix it. [NS05-NP]

For some participants, it was meaningful having even one person looking out for them to ensure they were taking personal time to prevent feeling overwhelmed: “*my boss is really good at checking in … and if I'm too tired, I'm going to take a morning off. Like they've never, never said anything”* [ON19-RPN].

Other participants attributed a sense of safety or security during the pandemic to the preparedness and support offered by the health setting in which they work:

I find that [the health authority] has probably given out a lot of education and then they also provided … some free counseling and then I have here, the [mobile health] app, they’ve partnered with [a corporate health entity] to provide free counseling for nurses as well. So, I have used that just a few times. More like, to debrief, right? [BC05-RN]

While some PCNs had these supports and worked in positive professional environments, other participants recognized that these were not universal experiences that everyone enjoyed, as public health emergencies can elicit a range of responses from leadership: “*COVID has certainly brought the best out in staff, it's brought the best out in management, but it's also brought out the worst”* [ON02-RN].

#### 3.2.3 Alignment of values and expectations

The smaller size of primary care teams, as well as the ability to manage patient flow and interactions in many primary care settings, provided PCNs with a sense of control over their work environment. However, steady leadership and an alignment of values within primary care settings and teams were necessary factors for participants that experienced that sense of control from their clinic setting. COVID-19 presented new concerns where values and expectations were sometimes misaligned:

… [family physicians] were safe working at home, right? So, I actually felt like a sitting duck to be honest because I’m immunosuppressed and the [clinic] protocols were not that strict. Like coming from working for [the health authority], where things were very strict… the fact that people are coming to work feeling unwell and their husband was at home with COVID, like, are you even kidding me right now? [BC04-RN]

Conversely, adherence to safety protocols mitigated the physical vulnerability PCNs felt in the context of a pandemic: “*So, I knew my workplace was safe because we did exactly what we were supposed to do. I knew that we were following the policies and procedures, I knew we were wearing our PPE appropriately”* [NS02-RN].

Participants also felt supported when their primary care setting was aligned in balancing patient care with COVID-19 precautions. As one participant noted, they felt unified with the rest of their clinic team when their suggestions (e.g., delaying non-urgent preventative care) were implemented:

… cases were up big time and we said, ‘we don't have to do this permanently but can we just … push these things off for a bit? Such as Paps’—it wasn't that we stopped doing Paps, but ‘can we put them off for a month until the surge goes up and comes back down again?’ And, again, I think it’s just because of my experience that they listen to me. [NS05-NP]

#### 3.2.4 Job control, flexibility, and autonomy

Some PCNs attributed the sense of control over their work and work environment with being in primary care:

Primary care, generally, our environments stayed very stable and we are very much in control of our own safety. So that puts us at ease … you usually work with a small group of likeminded people, so you can control your environment and feel very safe. So, whether it’s PPE or whether it’s just a place to go cry by yourself or whatever you needed, that was very much accessible. [ON02-RN]

Flexibility in their work environment allowed some participants to take time off, balance their family needs and schedules, and access wellbeing supports:

…in our organization, out of 13 people, there were five [staff] with young children and we made it work. So, whether that was alternating shifts like in-person vs. virtual, extended hours, working around the needs of people that were sick—everyone got COVID at least once. Just being realistic and also understanding that mental health is really important amongst healthcare providers. [ON05-RN]

Yet, PCNs were not always afforded the same level of autonomy over their work schedule as other members of their primary care clinics: “*if there was a time where I had to stay home, it was just [tough luck]”* [NL19-RN]. Another RN elaborated on the differences PCNs faced in providing patient care relative to family physicians (FPs) who were permitted to work remotely: “*the doctors were doing a lot of stuff by phone because people didn't want to necessarily come in, but I was still seeing people face-to-face all day long. And it was scary”* [NS01-RN].

#### 3.2.5 Rewards

Some participants felt a sense of satisfaction when the work they put in over the course of the pandemic had positive outcomes, such as effective protocols that prevented infections or strengthened nurse-patient relationships:

… seeing that there has been some positive outcomes from vaccinations, and people following those precautions and isolation protocols … just knowing that it worked was a good thing I think for all of us. Because some of our patients started to really trust in the fact that this matters, we're going to keep doing our part. And then you just didn't feel as defeated, I think. [ON05-RN]

The recognition of PCNs’ efforts by their supervisors or by other clinicians was also meaningful: “*the doctors realized how hard I was working … And after, when things opened up, they took me out for dinners, they didn't want me to leave … really going that extra mile to show their appreciation*” [NS09-RN].

Conversely, participants who did not see any positive outcomes or feel appreciated reported feeling deflated. An RN working in an under-resourced community with people experiencing marginalization expressed how difficult it was to see their patients continue to suffer despite their efforts, with community conditions (e.g., access to housing, toxicity of the street drug supply, population health) worsening during the pandemic:

I just feel like it’s this lack of any sort of movement or change in a positive direction. It feels like we’re actually going backwards … and so you feel like you’re just working so hard to keep your clients head just above the water and the water is rising so fast. [BC11-RN]

Other participants, feeling similarly defeated, wished there was greater acknowledgement for the roles they and their primary care colleagues filled throughout the pandemic. Namely, several participants would have liked to have received the same level of recognition (and compensation) as healthcare workers in acute care settings:

I feel that nurses, maybe in primary care as well, we did feel undervalued. So, I think we really did find that it hit home when pandemic pay went out, but we never got pandemic pay … it was almost like, well, we're not working as hard as everybody else or we're not in as much risk, but we were. [ON21-RPN]

#### 3.2.6 Professional relationships and social support

PCNs relied on their professional relationships to help manage the stresses of their various pandemic responsibilities. Some of these supports contributed toward PCNs’ professional practice, like a group message for NPs in a region to stay current on protocols, helping to sort through the constant updates and changes in procedures. Other supports helped PCNs manage their personal responsibilities by rearranging shifts or alternating between in-clinic and remote work to be able to stay home with children.

Those professional supports were integral to PCNs, in part because of the shared experiences to which participants felt others outside their work could not relate:

I wouldn't say there was a lot of support other than each other because people are so good in the nursing profession to work as a team. And I think there was so much uncertainty. No one really knew what was going on and what our world was like, except for the people that were there. So, people were really understanding of each other and very supportive. No one questioned sick time. People were very worried about each other all the time because it almost felt like we didn't have support outside of each other. [NS07-NP]

Though there had been an outpouring of support for healthcare workers early in the pandemic, over time that shifted to outward expressions of opposition to the pandemic response and those involved in it. One RN recounted how they became a target of social hostilities:

… a man did make sheep sounds towards me. And I said, ‘Excuse me?’ and he started, you know, ‘baa’-ing at me again and saying that we were a bunch of idiots. And I walked away and got into an office with a locked door. [BC09-RN]

#### 3.2.7 Work-life integration

Finding the right balance of work-life integration was challenging for participants. Working from home using virtual modalities to connect with patients could lead to a blurring of boundaries between home and work: “*I certainly did experience burnout just because it felt like it was burning the candle at both ends for quite some time and especially with virtual not setting great boundaries”* [ON17-NP].

Throughout the pandemic, PCNs also noted how their professional experiences informed their vigilance against COVID-19 in their personal lives:

I was so rigid about my COVID protocol in my personal life because I'm a healthcare worker, my situation is different. I work with people who are immunocompromised, I work with people who are higher risk. I cannot take the chance of doing something in my personal private time that would jeopardize my patients. [NS01-RN]

This fear of transmitting COVID-19 was bi-directional, with participants worried about transmitting the virus both to patients and family members. Decisions to minimize social interactions in light of these concerns, however, left participants feeling isolated and missing out on key relationships, for years in some cases: “*it was lonely because you can't see your kids or your grandbabies. I missed almost two years of my grandkids…”* [ON18-RPN].

## 4 Discussion

We thematically analyzed interviews with 76 PCNs in four regions of Canada to explore their personal and professional experiences during the COVID-19 pandemic. Applying the National Academy of Medicine's systems model of clinician burnout and professional wellbeing (29) to understand how the pandemic changed participants’ experiences of job demands and resources, we found that the demands placed on PCNs increased during the pandemic in nearly every category. At the same time, the pandemic altered job resources that might normally protect against burnout. That is, benefits in PCNs’ professional lives such as their sense of autonomy, social support, and work-life integration diminished as their workload, workplace stressors, and experiences of moral distress increased.

Participants consistently raised excessive workload and inadequate staffing concerns. Echoing the pandemic experiences of FPs and healthcare professionals in other (non-primary care) settings and countries (11, 12, 25, 39–42), PCNs in Canada frequently described long hours and consecutive days worked as well as an inability—both perceived and actual—to take time off. In part, inadequate staffing to support PCNs, both at work and when they needed to step away to recuperate, reflect primary care workforce issues that predated the COVID-19 pandemic (43).

Isolation requirements following COVID-19 exposure or illness led participants to feel guilty due to the burden their absence would place on colleagues or on patients unable to receive timely care—another experience shared by FPs (18). Yet, where many FPs were afforded a certain flexibility through the rapid expansion of virtual modalities to continue providing care for their patients if they had to isolate following a COVID-19 exposure, not all PCNs had this option due to limitations on their autonomous practice, differences in patient care roles and clinic settings, and/or employer-imposed restrictions (44). Conversely, PCNs who continued to work remotely during an illness via virtual care may not have had a much-needed reprieve from work. Moreover, the rapid expansion of virtual care during the pandemic in Canada introduced additional dimensions of technostress (45–47) for PCNs who could be required to navigate multiple interfaces in their own clinical practice and in coordinating patient care with other providers.

Experiences of guilt and moral distress were frequently tied to participants’ fears of bringing their workplace exposures home with them and were commonly articulated by participants who provided in-person care throughout the pandemic, sometimes with inadequate PPE. These are consistent with previously documented experiences of PCNs internationally (15, 16, 27, 28), FPs in Canada (14, 17, 18, 42, 48) and internationally (12, 40, 49), as well as other healthcare professionals (41, 50–52).

PCNs continued to work throughout the pandemic despite the multitude of challenges; this commitment was frequently attributed to a professional responsibility, recognition of the need for PCNs’ skillset in the pandemic response, and/or the belief that their contributions were beneficial. This connects to existing research that points to the vocational calling that motivates healthcare professionals to push through fatigue and stress but which, alone, cannot prevent them from experiencing burnout (29, 53). While several participants pointed to the primary care environment as being one which lends itself to greater control thanks to smaller team sizes and an ability to manage patient flow and interactions, other participants’ experiences suggest that this only holds true where primary care settings and staff are aligned in their implementation of public health precautions and clinic policies. In this respect, PCNs—particularly RNs and LPN/RPNs given their limited clinical and administrative autonomy in primary care—may be dependent on physician and health system employers to set policies, procedures, and a workplace culture that ensures the health and wellbeing of all staff.

Multiple participants from ON also pointed to their disparate experiences of rewards and appreciation relative to health professionals working in acute care settings, namely with respect to pandemic compensation. Similar to the provision of pandemic-related supports, pandemic pay was intended for “frontline staff” in hospital, residential care, social services, corrections and community settings, yet primary care was not explicitly mentioned in the workplace eligibility descriptions (54, 55). Combined with a lack of primary care supports, this added to a perception among these participants that their contributions to the pandemic response were not valued even if they recognized how much they had risked and given of themselves.

Participants’ pandemic experiences—both in the demands they faced and the resources they relied upon—provide an indication of the supports PCNs need to help mitigate against burnout and support their wellbeing during a pandemic or other times of heightened health system strain. While an emergent virus is likely to introduce new sources of stress in PCNs’ professional lives through changes to their workflow and administrative processes and exacerbate their workload, primary care clinics and employers can mediate the effects of these demands on PCNs. The National Academy of Medicine's systems model of clinician burnout and professional wellbeing recognizes that decisions and actions occurring across the health system—from frontline care delivery to the environment in which the health workforce operates—contribute to clinician burnout (29).

Better pandemic preparedness, including information suited specifically to primary care settings (such as infection protection and control procedures, guidelines for providing in-person care, addressing vaccine hesitancy), organized locum coverage, availability of PPE, and judicious redeployment of primary care workers to preserve primary care capacity are needed to promote PCN wellbeing (18, 56–61). Moving beyond the pandemic, as regular health services resume and primary care providers contend with catching up on delayed and deferred preventive, routine, and elective care (62–65) and virtual care persists (44, 66, 67), PCNs will need continued system supports to help manage ongoing heightened job demands, particularly amidst a diminished primary care workforce (24, 68) and patients experiencing increased clinical complexity (65, 69). This requires that health systems recognize the demands on primary care, inclusive of providing primary care surge capacity (57, 60, 63) and training and guidelines (e.g., triage, practice guidelines) focused on virtual care (44, 47, 67). Examining PCNs’ job demands and resources, including any preexisting vulnerabilities and how they were affected by a high-stress period such as a pandemic, provides insights for systems-based interventions toward improving PCN wellbeing and preventing burnout.

### 4.1 Limitations

We conducted interviews in four Canadian provinces; findings may not reflect the experiences of PCNs in other health jurisdictions or regions that experienced the COVID-19 pandemic differently. Further, given that interviews were conducted in the later stages of the pandemic in Canada, participants’ responses may be subject to recall bias (70). Though we applied a maximum variation sampling approach, we may not have fully captured all PCN experiences and perspectives. In particular, most of our participants identified as women, so our results may not be generalizable to other gender identities. Additionally, while our sample includes LPN/RPNs, RNs, and NPs, we did not observe any clear differences between these three nursing designations in our thematic analyses, suggesting an important area for future research to explore. Lastly, most of our participants were still working in primary care settings at the time of their interview. Accordingly, findings do not necessarily capture the experiences of PCNs who have left the primary care or health workforce as a result of their wellbeing.

## 5 Conclusion

The demands placed on PCNs increased during the pandemic while, at the same time, the resources that might normally protect against burnout decreased. The benefits from PCNs’ professional lives diminished as their workload, workplace stressors, and experiences of moral distress increased. Organizational culture, alignment of values and practices, and workplace autonomy were key to mitigating PCN burnout and stress. Better pandemic preparedness, including supports suited to primary care settings to promote physical and psychological safety, workflow efficiency, worker rest and recovery, and preservation of primary care capacity are needed to promote PCN wellbeing during a pandemic and other extended health emergencies.

## Data Availability

The datasets presented in this article are not readily available due to the inability to fully anonymize the data collected and analyzed for this research. De-identified data may be made available on request to the researchers, subject to approval from Simon Fraser Research Ethics (dore@sfu.ca), the Health Research Ethics Board of Newfoundland and Labrador (info@hrea.ca), Nova Scotia Health Authority Research Ethics Board (marie-laurence.tremblay@nshealth.ca), and Western University Research Ethics Board (res-serv@uwo.ca). Requests to access the datasets should be directed to lindsay_hedden@sfu.ca.
