# Supplementary material for: “You do it for the patient”: a qualitative analysis of changes to primary care nurses’ workplace demands and resources during the COVID-19 pandemic in Canada
Source: Front Health Serv. 2025 Jun 26;5:1557654. doi: 10.3389/frhs.2025.1557654 (PMC12241095; doi:10.3389/frhs.2025.1557654)
Supplement: Supplementary file 1 [file Datasheet1.pdf]

Interview Guide

In this study, we want to gain a better understanding of the roles of nurses (registered nurses, registered or licensed practical nurses and nurse practitioners) during a pandemic. We are interested in nurses who normally work in primary care settings. By roles, we mean specific tasks and/or responsibilities that nurses were asked or required to do during various stages of the pandemic.

The interview will be recorded and transcribed. You will not be identified in any report or presentation; your name will be replaced by a participant code and any identifying information will be obscured.

Participation is voluntary. You may end the interview at any time or choose not to answer any specific question. Any questions before we begin?

First, I would like to ask some general background questions.

1. How long have you been practicing as a registered nurse/licensed practical nurse/nurse practitioner?
2. In which communities do you currently practice and how would you describe them in terms of urban or rural?
3. What primary care setting do you normally work in? Who is your employer?
4. Can you tell me about the nature of your practice in primary care (before the pandemic), in terms of where you work and the nature of the services you provided?
5. Do you work in a single setting or are you split across multiple sites?
6. Beyond your clinical work as an [RN/LPN/NP], do you fill any defined roles in your clinic?
7. What is your gender?
8. Outside of your professional practice, do you routinely care for dependent family members?

In the next set of questions, I'd like to focus on the period from January to mid-March 2020. During this time, we first started to hear about COVID19 and cases were starting to show up in Canada. I'm going to refer to this the "pre-pandemic" period.

1. During the PRE-PANDEMIC period, could you describe what roles or functions you carried out related to COVID19?
   [Probe based on responses to first set of questions]: compare/contrast between different locations for nurses working in multiple settings (community- based practice, LTC, ED, hospital, other)

2. Can you tell me about any supports that were available to you to help carry out these roles? What barriers did you experience?

[Probe]: access to PPE, funding, communications with networks/groups/hospital
[Probe]: sufficient guidance/information to adapt your practice; which resources did you utilize and were they COVID-specific?

   a. How were these resources obtained? (provided by employer/colleague/ professional association or self-identified?)
      i. Most trusted resource?
      ii. Enough information? Too much?
      iii. Consistency of information across sources?

3. Looking back, are there other roles you think primary care nurses could have played during the PRE-PANDEMIC period? What supports would be needed to carry out those roles?
   [Probe]: roles at different facilities
   [Probe]: access to PPE, funding, communications with networks/groups/hospital

Now, let's consider the PHASED CLOSURE & REOPENING period, from mid-March 2020 to today.

4. During the PHASED CLOSURE & REOPENING periods, could you describe what roles or functions you carried out related to COVID19?
   [Probe]: roles at different facilities
   [Probe]: screening, testing

5. Can you tell me about what supports were available to you to help carry out these roles or any barriers you experienced?
   [Probe]: access to PPE, funding, communications with networks/groups/hospital
   [Probe]: sufficient guidance/information to adapt your practice; which resources did you utilize and were they COVID-specific?
   a. How were these resources obtained? (provided by employer/colleague/ professional association or self-identified?)
      i. Most trusted resource?
      ii. Enough information? Too much?
      iii. Consistency of information across sources?

6. Can you tell me about any experiences you had with redeployments during PHASED CLOSURE/REOPENING? That could include your own experience with being redeployed or any experiences/impacts that redeployments had on your work/workplace during these periods.
   [Probe]: how have redeployment(s) impacted patient care?

7. Looking back, are there other roles you think primary care nurses could have played during the PHASED CLOSURE & REOPENING period? What supports would be needed to carry out those roles?

[Probe]: roles at different facilities
[Probe]: access to PPE, funding, communications with networks/groups/hospital

Now, let's consider an ACUTE CARE CRISIS stage where a hospital may be overwhelmed by COVID cases.

8. Did your region ever enter an ACUTE CARE CRISIS stage? Can you tell me about it?
   [Probe]: when, where, how long did it last?

9. During the ACUTE CARE CRISIS, could you describe what roles or functions you carried out? OR If your region were to experience an ACUTE CARE CRISIS, what roles or functions could primary care nurses carry out?

10. Were you or any of the health care professionals with whom you work redeployed during this ACUTE CARE CRISIS and, if so, can you tell me about that experience?
   [Probe]: for how long/to do what?
   [Probe]: impact on patient care in regular clinical setting?

11. Can you tell me about what supports were/should be available to you to help carry out these roles?
   [Probe]: access to PPE, funding, communications with networks/groups/hospital

12. [Ask only if applicable] What barriers did you experience?
   [Probe]: access to PPE, funding, communications with networks/groups/hospital

13. [Ask only if applicable] Looking back, are there other roles you think primary care nurses could have played during the ACUTE CARE CRISIS? What supports would be needed to carry out those roles?
   [Probe]: roles at different facilities
   [Probe]: access to PPE, funding, communications with networks/groups/hospital

Now, I'd like to consider VACCINATION, which started in December 2020 and has been ongoing.

14. During the VACCINATION period, could you describe what roles or functions you carried out related to COVID19?
   [Probe based on responses to first set of questions]: screening
   Community-based practice, LTC, ED, hospital, other.

15. Can you tell me about what supports were available to you to help carry out these roles? What barriers did you experience?
   [Probe]: access to PPE, funding, communications with networks/groups/hospital

16. Can you tell me about any experiences you had with redeployments throughout the VACCINATION period? That could include your own experience with being redeployed or any experiences/impacts that redeployments had on your work/workplace during these periods.
    [Probe]: how have redeployment(s) impacted patient care?

17. Are there other roles you think primary care nurses could play during the VACCINATION stage? What supports would be needed to carry out those roles?
    [Probe]: roles at different facilities
    [Probe]: access to PPE, funding, communications with networks/groups/hospital

18. Looking back over these different periods, how did these pandemic related roles impact your regular job and nursing activities?

Now, looking forward, I'd like to consider the RECOVERY period once the pandemic is over. People suggest that there will be an increased demand for primary care services.

19. During the RECOVERY period, what roles or functions should primary care nurses carry out? How would these differ, if at all, from your normal pre-pandemic roles?

20. What supports should be available to you to help carry out these roles? What barriers do you anticipate?

For the final set of questions, I'd like to switch gears a bit.

21. Can you tell me about other (non-professional) roles and responsibilities you have in your life? For example, caring for children or other family members

22. How do these responsibilities influence the roles that you are able to play in a pandemic? What supports are needed to allow nurses with other responsibilities to fulfill pandemic roles? What barriers did you experience?

23. Thinking of your gender...Does your gender influence the roles that you are able to play in a pandemic? What supports are needed to allow all genders to fulfill these roles? What are the barriers?

Should we go through additional pandemic stages, may we contact you about doing another interview in the future about those additional stages and roles? You can decide whether you want to participate at that time.

Those are all the questions I have. Is there anything you would like to add?
